# Thermophysiological adaptations to passive mild heat acclimation

**DOI:** 10.1080/23328940.2017.1303562

**Published:** 2017-03-10

**Authors:** H. Pallubinsky, L. Schellen, B. R. M. Kingma, B. Dautzenberg, M. A. van Baak, W. D. van Marken Lichtenbelt

**Affiliations:** a Department of Human Biology and Movement Sciences, NUTRIM, Maastricht University, the Netherlands; b School of Built Environment and Infrastructure, Avans University of Applied Sciences, the Netherlands

**Keywords:** body temperature distribution, core temperature, heat adaptation, passive mild heat acclimation, thermal physiology

## Abstract

Passive *mild* heat acclimation (PMHA) reflects realistic temperature challenges encountered in everyday life. Active heat acclimation, combining heat exposure and exercise, influences several important thermophysiological parameters; for example, it decreases core temperature and enhances heat exchange via the skin. However, it is unclear whether PMHA elicits comparable adaptations. Therefore, this study investigated the effect of PMHA on thermophysiological parameters. Participants were exposed to slightly increased temperatures (∼33°C/22% RH) for 6 h/d over 7 consecutive days. To study physiologic responses before and after PMHA, participants underwent a temperature ramp (UP), where ambient temperature increased from a thermoneutral value (28.8 ± 0.3°C) to 37.5 ± 0.6°C. During UP, core and skin temperature, water loss, cardiovascular parameters, skin blood flow and energy expenditure were measured. Three intervals were selected to compare data before and after PMHA: baseline (minutes 30–55: 28.44 ± 0.21°C), T1 (minutes 105–115: 33.29 ± 0.4°C) and T2 (minutes 130–140: 35.68 ± 0.61°C). After 7 d of PMHA, core (T1: −0.13 ± 0.13°C, *P* = 0.011; T2: −0.14 ± 0.15°C, *P* = 0.026) and proximal skin temperature (T1: −0.22 ± 0.29°C, *P* = 0.029) were lower during UP, whereas distal skin temperature was higher in a thermoneutral state (baseline: +0.74 ± 0.77°C, *P* = 0.009) and during UP (T1: +0.49 ± 0.76°C, *P* = .057 (not significant), T2:+0.51 ± 0.63°C, *P* = .022). Moreover, water loss was reduced (−30.5 ± 33.3 ml, *P* = 0.012) and both systolic (−7.7 ± 7.7 mmHg, *P* = 0.015) and diastolic (−4.4 ± 4.8 mmHg, *P* = 0.001) blood pressures were lowered in a thermoneutral state. During UP, only systolic blood pressure was decreased (T2: −6.1 ± 4.4 mmHg, *P* = 0.003). Skin blood flow was significantly decreased at T1 (−28.35 ± 38.96%, *P* = 0.037), yet energy expenditure remained unchanged. In conclusion, despite the mild heat stimulus, we show that PMHA induces distinct thermophysiological adaptations leading to increased resilience to heat.

## Introduction

Heat acclimation studies typically report changes of physiologic parameters, for example of core and skin temperature and sudomotor functions, in rest as well as during exercise^1-8^ Different approaches of heat acclimation have been tested in the past, mostly to develop optimal heat adaptation models for miners, athletes or the military. It is generally believed that a relatively strong (heat) stimulus is needed to catalyze the anticipated changes, and therefore, most heat acclimation studies combine exposure to high ambient temperatures and exercise (active heat acclimation), to ensure the effectiveness of the intervention.^8^

Passive heat acclimation, i.e., without exercise, is a phenomenon likely to occur in everyday situations, for example due to prolonged occupancy of an overheated building,^9^ during a holiday in a warm country or simply during a warm summer or a heat wave. Considering the progress of global warming, the occurrence of those events is likely to be more frequent, even in European oceanic and humid continental climates (Köppen climate classification).^10-12^ However, only few studies have evaluated the effect of an external ‘passive’ heat stimulus on human thermophysiology alone,^13-20^ without additional elevated endogenous heat production.

Those earlier laboratory studies investigating passive heat acclimation applied, for example, a combination of heat exposure and vapor-barrier suits^13^ or hot water immersion^18-20^ to induce controlled hyperthermia. Other studies incorporated prolonged exposure to high ambient temperatures between 45˚C and 55°C.^15-17^ Such passive heat acclimation results significant reductions of core temperature and sweating and improved cardiovascular function, indicating increases the resilience to heat. A study in mice has, furthermore, shown that also prolonged passive exposure to a relatively mild ambient temperature (5 d, approximately 37°C) elicits physiologic changes such as a decreased core temperature during heat exposure.^21^ Human field studies show that naturally acclimatized Pima Indians have a lower sleeping core temperature than matched Caucasian counterparts.^22^ However, more structured information considering passive *mild* heat acclimation, without the induction of controlled hyperthermia and only induced by the exposure to warm ambient air is lacking.

Recently, it has been suggested that regular exposure to warmth might also have important implications for metabolic and cardiovascular health.^23^ In rats, it has been shown that heat treatment improves glucose tolerance ^24^ and shifts obesity-induced insulin resistance back to normal, healthy insulin sensitivity.^25^ Heat therapy by means of water immersion in young healthy volunteers has been shown to significantly improve cardiovascular function.^19,20^ However, it is not yet known if exposure to warm ambient air elicits comparable health effects in humans. It is therefore of particular interest to study the effects of passive heat acclimation on energy metabolism and cardiovascular parameters, as it might help to understand how temperature exposure could possibly contribute to the treatment of metabolic and cardiovascular disorders.

Considering the very limited knowledge on the effects of passive mild heat acclimation on thermophysiology in humans, the main objective of the present study was to evaluate the effect of passive mild heat acclimation on core temperature, skin temperature, water loss, cardiovascular parameters, energy expenditure and skin blood flow.

## Methods

This study was conducted in the period of December 2014 till August 2015. In this period, the average day outdoor temperature as recorded 2 weeks previously to the start of each individual measurement ranged between 1.5°C and 20.2°C (11.5 ± 7.1°C mean ± SD). The Medical Ethics Committee of Maastricht University approved the study and it was conducted conform the Declaration of Helsinki (Fortaleza, Brazil, 2013).

### Participant characteristics

In total, 11 healthy male Caucasian volunteers participated in the study Table 1). All participants were normotensive, non-obese, non-smokers and did not take any medication that might have influenced the thermoregulatory system. Before commencing the study, all participants were provided with detailed information regarding the purpose and the methods of the study. All gave written informed consent.

**Table 1.** Participant characteristics.

	Mean	± SD
Age [years]	24.6	2.7
Height [m]	1.79	0.07
Weight [kg]	72.2	8.9
BMI [kg/m^2^]	22.6	2.9
Fat percentage [%]	19.7	3.0
Fat mass [kg]	14.5	3.3
Habitual physical activity [Baecke score total]	8.1	1.4

*N* = 11, data is presented as mean ± SD.

### Study design

**Figure 1.**
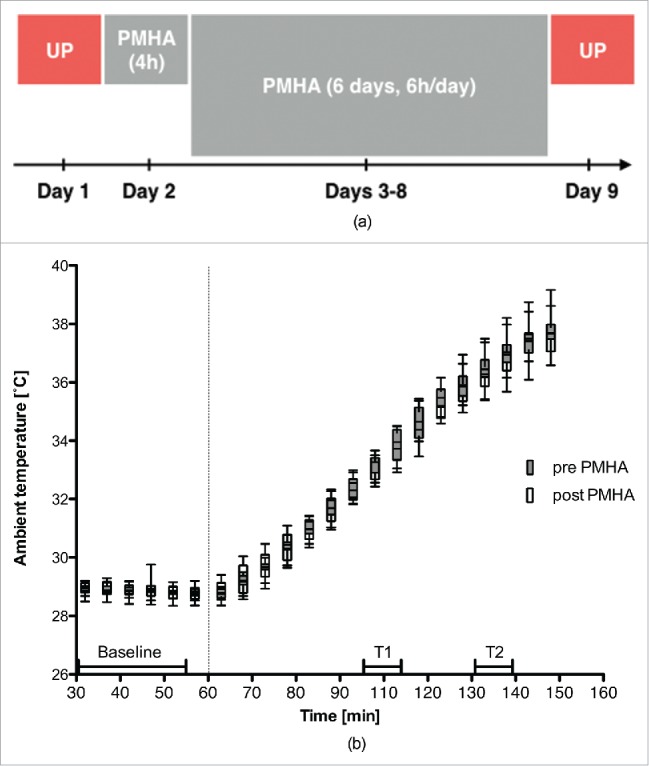
(A) Overview of the study procedures. PMHA refers to passive mild heat acclimation. UP refers to the temperature ramp protocol as described in the methods section. During UP, participants were exposed to an incremental ambient temperature ramp (28.8±0.3°C to 37.5±0.6°C). (B) Experimental conditions during protocol UP (*n*=11). Each boxplot represents a 5-min interval of the ambient temperature after the start of the temperature drift. Whiskers indicate ±1 SD. Baseline (28.44±0.21°C mean ± SD), T1 (33.29 ± 0.4°C) and T2 (35.68 ± 0.61°C) represent the respective intervals used for data analysis.

Participants were exposed to 7 d of mild passive heat acclimation (PMHA) (Fig. 1A). To study the physiologic response to high temperatures, participants underwent an increasing temperature ramp before and after PMHA, which will be referred to as UP (Fig. 1A and B).

#### UP protocol

For protocol UP, participants arrived at the laboratory in the morning after an overnight fast (as of 22:00h). Both evenings before the UP measurements took place, participants consumed a self-chosen standardized evening meal.

**Figure 2.**
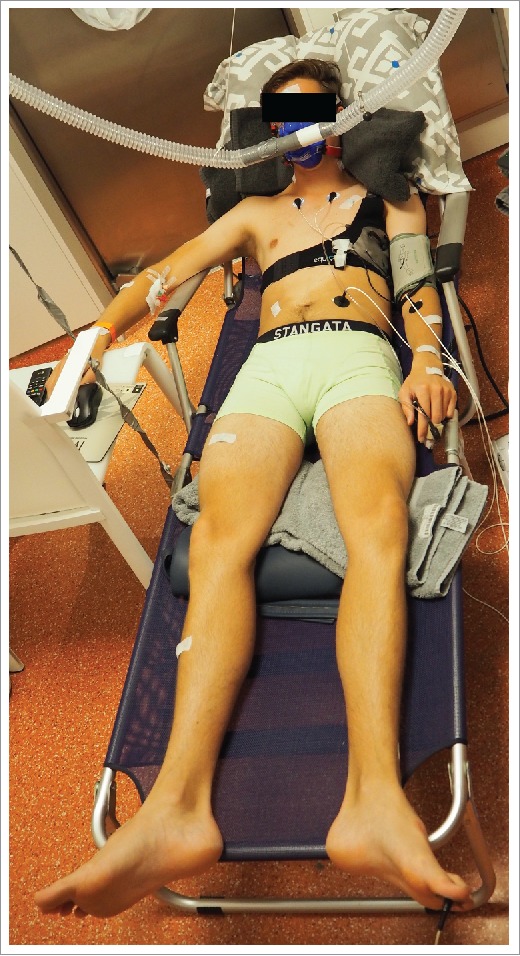
Participant during UP in a climate chamber.

UP started with a baseline period of 60 min at 28.8 ± 0.3°C (Fig. 1B). The baseline temperature was assumed to be the neutral temperature for a resting semi-nude person, based on the literature review of Kingma et al.^26^ and it was adjusted for the isolation of the stretcher that participants lay on during the measurements (Fig. 2). After the baseline period, surgical temperature increased over the course of 90 min to 37.5 ± 0.6°C (Fig. 1B). Relative humidity drifted with changes in temperature, resulting in an average relative humidity of 25.8 ± 7.2% during UP. Three time intervals were selected to compare data before and after PMHA (protocol time and ambient temperature in brackets): baseline (minutes 30–55: 28.4 ± 0.2°C), T1 (minutes 105–115: 33.3 ± 0.4°C) and T2 (minutes 130–140: 35.7 ± 0.6°C) (Fig. 1B).

#### Physiological measurements

Upon arrival at the laboratory, participants ingested a telemetric pill (Vital Sense, Philips Healthcare, NL) to measure core temperature. To detect the signal of the telemetric pill, an Equivital apparatus was attached to the participant's body using a chest strap (Equivital Hidalgo, UK). The same device was used to record heart rate. To calculate mean skin temperature, wireless skin temperature sensors (iButton, Maxim Integrated Products, California, USA) were attached to 14 ISO-defined body sites^27^ with semi-adhesive tape (Fixomull stretch, BSN medical GmbH, GER). Proximal skin temperature was calculated as an average of the ISO-defined sites of scapula, low back paravertebral, upper chest and abdomen. For the distal skin temperature, skin temperatures of hand and instep were averaged. Core temperature, heart rate and skin temperatures were recorded at 1-minute intervals.

In the climate chamber, participants took place on a stretcher with air-permeable fabric (Fig. 2). Here, Laser Doppler Flowmetry (LDF) probes were fixated to the participant's thenar and ventral side of the underarm halfway between carpus and antebrachium, to continuously measure skin blood flow (10Hz; PeriFlux System 5000, Perimed, SE). A finger blood pressure cuff was attached to assess cardiac output (CO) (Finometer MIDI, Amsterdam, NL) at baseline, T1 and T2, and upper arm blood pressure was measured at the same time points on the other arm by auscultation (Medisana MTP, Medisana AG, GER). Energy expenditure was continuously measured using indirect calorimetry with a respiratory gas analyzer. Metabolic rate was calculated using the method of Weir^28^ from the consumption of oxygen and the production of carbon dioxide. Immediately before entering the climate chamber and after leaving it, participants were weighed to determine total water loss, using the difference in total body mass before and after the UP protocol.

#### Passive mild heat acclimation

PMHA commenced in the noon of study day 2 (Fig. 1A). During the first sequence of PMHA, participants stayed in a “warm chamber” for 4 h. During the remaining 6 d of PMHA, participants acclimatized for 6 h per day. From earlier active heat acclimation studies, we know that the most important changes are expected to occur within the first 4–6 d of heat exposure. Therefore, we applied a 7-d acclimation protocol.^29^

The operative temperature in the warm chamber was kept constant at 33.3 ± 1.6°C; and the relative humidity was 22.3 ± 6.6%, which classifies the ambient air as dry. All participants successfully completed the passive mild heat acclimation period.

During their stay, participants remained seated at a desk and were allowed to perform regular office work (1.2METs). Participants wore standardized clothing composed of underwear, T-shirt, shorts and socks/slippers. The total thermal resistance of the clothing ensemble plus the desk chair added up to approximately 0.41clo.^30,31^ Participants had unlimited access to water; and food was provided upon request, to not influence habitual diet. Participants were allowed to leave the warm chamber for toilet breaks.

### Data analysis

The software package PASW Statistics 22.0 for Mac (SPSS, Inc.) was used for the statistical analysis.

#### Physiological data

The first 30 min of protocol UP were regarded as familiarization period, and therefore excluded from the data analysis. For the comparisons of core temperature, skin temperatures, energy expenditure and skin blood flow within each of the protocols and before and after PMHA, three periods were selected during UP: baseline (*t* = 30–55 min 28.81 ± 0.40°C), T1 (*t* = 105–115, 34.81 ± 0.50°C) and T2 (*t* = 130–140, 37.53 ± 0.58°C). Energy expenditure was normalized for body surface area (m^2^). Since SkBF data were obtained in arbitrary units, the data measured during UP has been averaged per minute and has been analyzed relative to the baseline period.

Paired-sample *t*-tests were used to compare the measured parameters before and after PMHA. Repeated Measures ANOVA was performed to test for significant changes within the protocols (from baseline to T1 to T2). If the Assumption of Sphericity for the general linear model was violated, Bonferroni correction was applied as a post-hoc test. Linear regression analysis was performed to test the potential influence of outdoor temperature on the outcome parameters. Statistical significance was considered for *P* ≤0.05 and a statistical trend was considered if 0.05 < *P* <0.10.

## Results

### Core temperature

**Figure 3.**
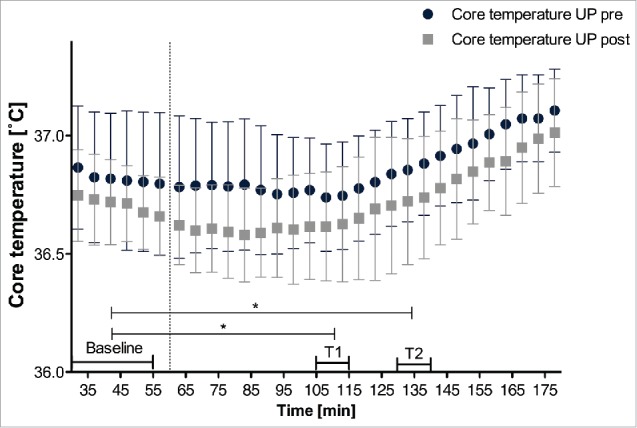
Average core temperature during protocol UP pre and post PMHA. T1 and T2 represent the respective intervals used for data analysis. Data is presented as mean±SD. *N*=11, *P < 0.05 for changes after PMHA.

After PMHA, core temperature was significantly lower during protocol UP at T1 (−0.13 ± 0.13°C, *P* = 0.011) and T2 (−0.14 ± 0.15°C, *P* = 0.026) Table 2 and Fig. 3), but not at baseline (−0.12 ± 0.23°C, *P* = 0.115).

**Table 2.** Body temperatures during UP pre- and post-PMHA.

Protocol UP	Baseline pre	T1 pre	T2 pre	Baseline post	T1 post	T2 post
Core temperature [°C]	36.80±0.27	36.74±0.25	36.87±0.22	36.72±0.18	36.62±0.23^*^	36.73±0.26^*^
Mean skin temperature [°C]	33.89±0.50	35.08±0.42	35.72±0.37	33.97±0.30	35.08±0.30	35.79±0.25
Proximal skin temperature [°C]	34.41±0.49	35.60±0.35	36.18±0.28	34.30±0.43	35.38±0.38^*^	36.03±0.32
Distal skin temperature [°C]	32.44±0.75	32.11±0.52	34.97±0.52	33.18±0.55^**^	34.60±0.49^$^	35.48±0.46^*^
Proximal–distal skin temperature gradient [°C]	1.97±0.83	1.49±0.64	1.21±0.61	1.12±0.66^*^	0.78±0.58	0.54±0.53^**^
Core–distal skin temperature gradient [°C]	4.40±0.81	2.64±0.59	1.90±0.61	3.54±0.51^*^	2.02±0.49^*^	1.29±0.55^**^

Data is presented as mean ± SD. *N*=11.

$ 0.05 < *P* < 0.1 for changes post-PMHA,

* *P* < 0.05 for changes post-PMHA,

** *P* < 0.01 for changes post-PMHA.

### Skin temperatures

After PMHA, average mean skin temperature was not significantly different from the pre measurement at any time point. However, average proximal skin temperature significantly decreased at T1 (−0.22 ± 0.29°C, *P* = 0.029) and average distal skin temperature increased at baseline (+0.74 ± 0.77°C, *P* = 0.009), and T2 (+0.51 ± 0.63°C, *P* = 0.022) and tended to be higher at T1 (+0.49 ± 0.76°C, *P* = 0.057) upon warmth exposure during UP post PMHA Table 2). Moreover, the gradient between average proximal and average distal skin temperature was significantly reduced after PMHA at baseline (−0.84 ± 0.94°C, *P* = 0.014), T1 (−0.71 ± 0.79°C, *P* = 0.014) and T2 (−0.67 ± 0.49°C, *P* = 0.001). The same was observed for the gradient between core temperature and distal skin temperature at baseline (−0.86 ± 0.84°C, *P* = 0.007), T1 (−0.61 ± 0.74, *P* = 0.021) and T2 (−0.56 ± 0.54°C, *P* = 0.009).

### Total water loss

Total water loss during UP decreased from 217.3 ± 62.1 ml before PMHA to 186.81 ± 50.18 ml after PMHA (*P* = 0.012).

### Cardiovascular parameters

Heart rate, systolic and diastolic blood pressure, stroke volume and cardiac output were measured at baseline, T1 and T2 during UP, before and after PMHA. At baseline, both systolic and diastolic blood pressure were significantly lower after PMHA (systolic: Δ −7.7 ± 7.7 mmHg, *P* = 0.015; diastolic: Δ −4.4 ± 4.8 mmHg, *P* = 0.001, Table 3). At T2 of UP post, systolic blood pressure was significantly lower than before PMHA (*P* = 0.003), but diastolic blood pressure was no longer significantly different from the pre measurements (*P* = 0.235, Table 3). Heart rate, stroke volume and cardiac output were not significantly affected post PMHA.

**Table 3.** Systolic and diastolic blood pressure pre- and post-PMHA.

Pre	Baseline	T1	T2
Systolic [mmHg]	118±9	117±11	118±9
Diastolic [mmHg]	72±7	70±10	67±9
Heat rate [bpm]	68±17	70±18	72±16
Stroke volume [ml]	93±17	94±18	82±30
Cardiac output [l/min]	5.9±1.5	6.3±1.7	6.3±1.7
Post	Baseline	T1	T2
Systolic [mmHg]	113±7^*^	113±9	113±8^*^
Diastolic [mmHg]	68±6^*^	66±7	64±6
Heat rate [bpm]	61±9	63±10	64±9
Stroke volume [ml]	96±11	92±12	94±14
Cardiac output [l/min]	5.8±1.4	6.0±1.4	6.3±1.3

Data is presented as mean ± SD. *N*=11,

* *P* < 0.05 for changes post-PMHA.

### Energy expenditure

To assess the effect of the temperature drift on energy expenditure, baseline values were compared with T1 and T2 Table 4). During the pre-measurement, energy expenditure tended to increase from baseline and T1 (+0.17 ± 0.26 kJ/min, *P* = 0.056) and significantly increased from baseline to T2 (+0.25 ± 0.20 kJ/min, *P* = 0.002). After PMHA, energy expenditure increased significantly from baseline to T1 (+0.19 ± 0.24 kJ/min, *P* = .024) and a trend was evident for baseline to T2 (+0.20 ± 0.31 kJ/min, *P* = 0.056).

**Table 4.** Energy expenditure and hand SkBF pre- and post-PMHA.

	Baseline pre	T1 pre	T2 pre	Baseline post	T1 post	T2 post
Energy expenditure UP [kJ/min]	4.83±0.55	5.00±0.63	5.07±0.59^*^	4.79±0.73	4.97±0.84^*^	4.99±0.85
Relative hand SkBF UP [%]	1.00±0.00	1.30±0.36^*^	1.67±0.68^*,#^	1.00±0.00	1.02±0.21^§^	1.40±0.60^*^

Data is presented as mean±SD, *N*=11.

* *P* < 0.05 for changes compared to baseline within the same protocol;

# *P* < 0.05 for changes from T1 to T2 within the same protocol.

§ *P* < 0.05 for differences between pre- and post-PMHA.

To determine the effect of PMHA on energy expenditure, baseline, T1 and T2 were compared before and after PMHA. No significant changes, and thus no effect of PMHA on basic metabolic rate (baseline) and energy expenditure were observed.

### SkBF

Before PMHA, there was a significant increase from baseline to T1 (± 36.83 ± 11.66%, *P* = 0.01), baseline to T2 (± 74.18 ± 21.37%, *P* = 0.006) and from T1 to T2 (+37.35 ± 12.63%, *P* = 0.014). After PMHA, the increase of SkBF was only significant between baseline and T2 (+49.00 ± 18.74%, *P* = 0.026; Table 4).

Post-PMHA, hand SkBF decreased significantly by 28.35 ± 38.96% (*P* = 0.037) at T1, but at T2, the decrease was no longer significant (*P* = 0.208).

## Discussion

This study evaluated the effects of passive mild heat acclimation (PMHA), i.e., without exercise, on human thermophysiology. PMHA is of particular interest, as it represents temperature challenges encountered in everyday life, which are fundamentally different to those studied with active heat acclimation (AHA). Whereas AHA addresses the effect of exogenous and endogenous heat stimuli, PMHA focuses only on relatively mild exogenous heat stimulus. We show that PMHA consisting of exposure to ∼33 °C at 7 consecutive days indeed elicited a decrease of core temperature and a redistribution of skin temperature in warm ambient temperatures. Water loss and blood pressure were decreased post acclimation. Energy expenditure, however, was not affected by PMHA.

### Core temperature

PMHA resulted in a decrease of core temperature during warming (−0.13°C at T1 (*P* <0.01) and −0.14°C at T2 (*P* = 0.026). This result is in line with many earlier studies that evaluated various models of heat acclimation.^6,13,14,32-35^ Although the core temperature decrease was often more outspoken in studies inducing controlled hyperthermia (e.g., approximately −0.19°C, orally measured core temperature.^35,36^), especially after acclimation to humid heat (e.g., ranging from −0.1 to −0.5°C^6^) and after active heat acclimation (−0.3°C to −0.4°C lower resting core temperature^33,37^), our results show that 7 d of PMHA also modulates core temperature. The observed decrease of core temperature combined with the increase of distal skin temperature also resulted in a reduced core-distal skin gradient post acclimation. The latter represents an effective adaptation mechanism for warm environments: a smaller temperature gradient between core and distal skin helps to create a certain thermoregulatory ‘buffer’, as the total tissue temperature increases more slowly^8,38,39^

### Skin temperature

There was no significant effect of PMHA on the course of mean skin temperature during warming, which is not in line with earlier findings from an AHA study.^33^ However, skin temperature distribution changed significantly: proximal skin temperature was decreased during warming, whereas distal skin temperature was increased during baseline and warming. As indicated above, the decreased temperature gradient between core and skin has the potential to decelerate the warming of the body. Additionally to the advantageous effect of higher distal skin temperatures on body warming, increased skin temperature also influences cutaneous water vapor pressure, which in turn facilitates evaporative cooling.^40^ Hence, the increase of distal skin temperature together with the decreased core temperature as found in the present study, represent important functional adaptation to heat.

### Water loss

After 7 d of PMHA, total water loss, as measured by the change of body mass before and after warming, was significantly decreased.

Changes of sudomotor functions after (short- or mid-term) active heat acclimation are commonly reported, indicating an increase of sweating capacity and an increased sudomotor sensitivity.^8,41^ The decreased total water loss, which was evident in the present study, might, however, suggest the contrary, namely a reduction of evaporative heat loss due to mild heat acclimation. The reason for this result might be due to the applied methods: The heat stimulus during the acclimation period was kept constant and the temperature increments during the pre- and post-tests were identical. Considering the lowered core temperature after PMHA, it can be concluded that a certain level of heat habituation had, indeed, been acquired. As a consequence, the same exogenous heat stimulus during UP after PMHA became less severe than it was before PMHA. Thus, less evaporative heat loss and sweating is needed to maintain the target core temperature, which might explain why the total water loss after acclimation was less.^8^

In the present study, no hydration assessment was performed to ensure comparable hydration status before and after PMHA, which might be considered as a limitation. However since the decrease of water loss post PMHA was clearly significant (P = .012), and participants served as their own controls, a confounding influence of hydration status is regarded as relatively unlikely.

### Cardiovascular parameters

In a thermoneutral condition (baseline), both systolic and diastolic blood pressures were significantly decreased after PMHA and systolic blood pressure was also significantly decreased during warming. Heart rate, cardiac output and stroke volume were not affected by PMHA.

The regulation of blood pressure is challenged during heat exposure. Blood flow in the extremities remarkably increases in a warm environment (vasodilation), thereby decreasing total peripheral resistance and blood pressure. In contrast, blood flow to the extremities decreases to a minimum in a cold environment (vasoconstriction), which increases total peripheral resistance and increases blood pressure. There is not much literature available describing the effect of passive heat acclimation on blood pressure. Two studies did not find an effect of active heat acclimation on blood pressure, although they report distinct changes of heart rate, cardiac output and stroke volume.^42,43^ Both studies incorporated exercise training combined with heat acclimation; and acclimation effects were evaluated during exercise in a warm environment. Blood pressure values in a neutral thermal environment, however, were not reported. Recent studies by Brunt et al.^19,20^ found that cardiovascular function and blood pressure were remarkably improved after long-term passive heat therapy (daily hot baths over the course of 8 weeks). The decrease of blood pressure is in line with our findings, despite the fact that the acclimation strategy of the present study was less intense, much shorter and without the application of controlled hyperthermia.

### Skin blood flow

As expected, we found that a warming thermal environment caused vasodilation and thereby an increased SkBF at the hand. Contrary to our expectations, PMHA resulted in an average *reduction* of the SkBF increment during the UP protocol of approximately 28%.

A decrease of SkBF in the heat after heat acclimation has earlier been reported in the literature, when different ethnic groups and acclimatized indigenous people to unacclimatized groups were compared^44,45^ Roberts et al.^2^ and others^46^ also found that after exercise training and heat acclimation, SkBF decreased. However, due to the increase of skin temperature at the extremities, a corresponding increase of SkBF would have been expected after PMHA. Moreover, considering the lowered core temperature and the decreased core-distal skin temperature gradient, earlier heat loss via the skin is stimulated, which would suggest an enhanced SkBF as well.

In this study, we measured SkBF using a Laser Doppler Flowmetry apparatus. Measurements with this technique scale linearly with changes in underlying blood flow velocity and blood volume, producing an output of arbitrary units rather than absolute values. Since the angle of measurement relative to the skin blood flow can be different between measurements, a normalization of the data to baseline is required. However, the applied normalization of the data to baseline implies that it is not possible to detect an effect of heat acclimation on baseline flow measurements. In this study, distal skin temperature was significantly higher after PMHA, which is why we assume that the same was true for SkBF. However, due to the normalization procedure of the SkBF data, the anticipated increase of SkBF at baseline is not observable. Moreover, as the absolute level of skin blood flow after PMHA may have been closer to its maximal value, the relative effect of SkBF increase relative to baseline during the temperature increment might have been blunted due to the methodology. The latter could therefore also account for the decreased SkBF measured post PMHA.

### Energy expenditure

Whereas an energy expenditure increase observed during cooling can be attributed to non-shivering thermogenesis (probably by the activation of brown adipose tissue) and shivering thermogenesis (muscle tissue),^23,47-50^ it remains uncertain to which tissue or bodily function the increase of energy expenditure during warming related. Although the reabsorption of electrolytes during sweating is an ATP-consuming process, the energy required for sweating is very small compared with the overall increase in energy expenditure. Possibly, an increase of heart rate and ventilation due to hyperthermia, which has earlier been shown, might partly explain the increase of energy expenditure.^51,52^ However, an increase of heart rate was not detected in the present study and ventilation was not measured. Another explanation for the increase of energy expenditure might be the Q10 effect.^53^ According to the Arrhenius law, a 1 °C change in mean body temperature, might account for an increase of the energy expenditure of as much as 8% (assuming Q10-factor = 2.3).^54^ However, we only find this relation between the Q10 effect and the percentage of change of energy expenditure between baseline and T1 post PMHA (*r* = 0.610, *P* = 0.046). Presumably, an increase of energy expenditure with an increasing ambient temperature is due to a combination of several factors, but the exact magnitude and mechanism of the energy expenditure increase remains uncertain.

With respect to the effect of PMHA on energy metabolism, we did not detect an effect of PMHA on the course of energy expenditure. Generally, a slight decrease of metabolic heat production in a warm environment is very advantageous, as it concurs with a decreased need for (evaporative) body cooling. Earlier (field) researches found a relation between heat acclimation and lower energy expenditure,^55-57^ which is likely to represent an adaptation due to more long-term or more intensive (active) heat acclimation. Contrarily, a controlled study comparing heat acclimatized Pima Indians with matched Caucasians did not detect a difference of basal metabolic rate.^22,58^ It is, however, difficult to differentiate between actual metabolic adaptations and changes resulting from modified thermoregulatory behavior.

### Limitations and future perspectives

Due to practical reasons, the present study was conducted over the course of 9 month between December 2014 and August 2016. As mentioned in the methods section, the mean day outdoor temperature, recorded 2 weeks before the start of each individual measurement, during this period varied between 1.5 °C and 20.2 °C (11.5 ± 7.1 °C mean ± SD). To rule out possible confounding, we used linear regression analysis to test if the mean outdoor temperature influenced the effect of PMHA on the outcome parameters (data not shown). However, no significant effects of season were detected.

Since we have found significant effects of PMHA on thermophysiological parameters after a short intervention period of only 7 d, more research is warranted to evaluate the long-term effects of prolonged warmth exposure and its potential decay on human thermophysiology. Moreover, it is of great relevance to evaluate the impact of PMHA on other health parameters such as insulin sensitivity and other cardiovascular related parameters in populations with metabolic and cardiovascular risk factors.

## Conclusion

This study evaluates the effect of passive mild heat acclimation (PMHA) on thermophysiology in humans. We show that PMHA induces adaptations of the human thermoregulatory physiology and cardiovascular system, leading to an improved resilience to warm ambient conditions. Energy metabolism is not affected by PMHA.

## Abbreviations

PMHA: passive mild heat acclimation
SkBF: skin blood flow
T1: time interval 1 used for data analysis as described in Methods section
T2: time interval 2 used for data analysis as described in Methods section
UP: temperature ramp protocol as described in Methods section
